# Hilar cholangiocarcinoma patients accepting preoperative percutaneous transhepatic biliary drainage experienced high incidence of portal vein invasion and lymph node metastasis

**DOI:** 10.3389/fonc.2025.1504604

**Published:** 2025-06-06

**Authors:** Yingke Cai, Yuxuan Yao, Yi Dong, Dang Wang, Jing Luo, Gang Heng

**Affiliations:** ^1^ Department of Hepatopancreatobiliary, Hernia and Vascular Surgery, Caidian District People’s Hospital of Wuhan, Wuhan, China; ^2^ Department of General Surgery, People’s Liberation Army of China (PLA) Middle Military Command General Hospital, Wuhan, China; ^3^ Department of Urology, General Hospital of Xinjiang Military Command, Urumqi, China

**Keywords:** HCCA, PTBD, portal vein invasion, lymph node metastasis, survival

## Abstract

**Background:**

Percutaneous transhepatic biliary drainage (PTBD) was widely used for bile drainage in hilar cholangiocarcinoma (HCCA) patients, due to its exact effectiveness in relieving obstructive jaundice. However, the potential association between PTBD and increased local tumor spread (including portal vein invasion and lymph node metastasis) remained unclear, as this procedure might prolong the waiting time and lead to potential risks of portal vein injury. This study aimed to investigate whether HCCA patients undergoing PTBD exhibit higher risks of portal vein invasion and lymph node metastasis after radical resection.

**Methods:**

The clinical data of 341 HCCA patients was retrospectively analyzed. PTBD was exclusively used as the preoperative biliary drainage method, excluding patients who underwent endoscopic nasobiliary drainage or endoscopic biliary stenting. Portal vein invasion and lymph node metastasis were verified by postoperative pathological examinations.

**Results:**

In this study, 163 patients (47.8%) received preoperative PTBD. These patients experienced significantly higher risks of portal vein invasion [odds ratio (OR): 1.86, p = 0.027] and lymph node metastasis (OR: 1.94, p = 0.008) compared to those 178 patients (52.2%) in the non-PTBD group. The Kaplan–Meier survival analysis revealed significantly better OS (p = 0.039) in the non-PTBD group. Causal mediation analysis revealed that the effect of PTBD on survival was partly mediated by portal vein invasion and lymph node metastasis. Additionally, the length of hospitalization in PTBD group was obviously longer (26.7 days vs. 21.8 days, p = 0.002).

**Conclusion:**

Preoperative PTBD was associated with increased incidence of portal vein invasion and lymph node metastasis in HCCA patients accepting R0 resection.

## Introduction

Hilar cholangiocarcinoma (HCCA) is a rare but highly aggressive malignant tumor originating from the extrahepatic bile duct (1). Preoperative percutaneous transhepatic biliary drainage (PTBD) has been extensively employed to relieve obstructive jaundice in HCCA patients prior to surgery, which can reduce the severity of liver damage, promote the ability of liver regeneration and increase the remaining liver volume (2–4).

Previous investigations have predominantly focused on the benefits of PTBD in reducing jaundice and enhancing hepatic function (5, 6). These studies confirm that PTBD effectively decreases serum bilirubin levels and optimizes the patients’ clinical status, thereby improving eligibility for curative-intent surgery (7). Current evidence regarding PTBD-related oncological complications has primarily focused on implantation metastases, including pleural and peritoneal dissemination (8–10). However, the potential injury of portal vein branches during catheter insertion might lead to portal vein thrombosis, with a reported incidence of 10% in prior research (11–13). Furthermore, the injury of portal vein might lead to the local spread of tumor cells from bile duct to portal veins, causing the invasion of related vessels. Moreover, the prolonged intervals between PTBD and operation might increase the incidence of local tumor invasion, such as vascular invasion and lymph node metastasis (10). Despite these insights, significant knowledge gaps persist regarding PTBD’s specific impact on local tumor invasion patterns, specifically vascular invasion and regional lymph node metastasis. Understanding whether PTBD contributes to local tumor spread is crucial for optimizing preoperative management strategies and improving patient outcomes.

Therefore, we conducted a retrospective analysis of 341 HCCA patients accepting radical resection to investigate the potential associations between PTBD and regional vascular invasion and lymph node metastasis. Also, the potential effects of PTBD on the overall prognosis and survival of HCCA patients were discussed. Through this investigation, our study aimed to inform clinical decision-making and refined the preoperative management of this challenging malignancy.

## Methods

### Study population

From March 2008 to December 2019, 341 HCCA patients undergoing radical resection at the Southwest Hospital and PLA Middle Military Command General Hospital were enrolled. All procedures were performed according to R0 resection criteria. This study was approved by the Ethical Committee (Approval No. KY20211215), and all patients or their guardians provided written informed consent when informed of potential risks or adverse effects during the clinical process.

### Data collection

In this study, preoperative epidemiologic features like age, gender, height, weight, BMI, heart disease and diabetes, laboratory tests like white blood cells, platelet, liver transaminase, bilirubin, albumin, alkaline phosphatase, coagulation and tumor markers, clinic-pathological features like liver cirrhosis, hepatic artery invasion, portal vein invasion, lymph node metastasis, tumor differentiation, and Tumor Node Metastasis (TNM) stage were collected.

The inclusion criteria of this study included the following: patients with pathological confirmation of HCCA; patients received R0 resection; expected survival time longer than 3 months; patients had a clear record of accepting preoperative biliary drainage or not; and PTBD was the unique approach of biliary drainage. This R0 resection implied the histologically negative margins in the operation. Moreover, the proximal end of the bile duct greater than 5 mm from the edge of the tumor and the distal end at the upper edge of the pancreas were resected in the operation. The entire caudate lobe and surrounding liver parenchyma greater than 15 mm surrounding the bile duct axis were removed. For patients with Bismuth–Corlette III or IV HCCA, hemihepatectomy or extended hemihepatectomy and caudate lobectomy were conducted. The lymph nodes of groups 8, 12, and 13 were conventionally dissected.

The criteria of accepting PTBD in this study included the following: the level of total bilirubin was higher than 100 µmol/L or the symptom of jaundice has sustained for more than 4 weeks, or those patients needed to accept large scale hepatectomy (over thee Couinaud segments).

These patients who accepted R0 resection did not routinely accept adjuvant therapy after operation. All individuals were closely followed up in outpatient clinic after discharge. Basic measurements including liver function and blood routine examination, examination of serum tumor markers, and imaging were performed.

### Statistical analysis

Continuous variables were presented as the mean and standard deviation or median and interquartile range. Categorical variables were presented as amounts and percentages. Comparisons between clinicopathological factors were made using Student’s t-test or analysis of variance or Kruskal–Wallis test for continuous variables and χ2-test for categorical variables. The log-rank test and t-test were used to compare the differences between those patients accepting PTBD or not.

Univariate and multivariate logistic regression were used to analyze the risk factors associated with portal vein invasion and lymph node metastasis. Odds ratios (OR) derived from logistic regression models represent the likelihood of pathological outcomes per unit increase in exposure, with p-values <0.05, indicating statistically significant associations. The Cox proportional-hazards model was used to identify relationship between clinical characteristics and survival. Kaplan–Meier curves were used to estimate overall survival (OS) and relapse-free survival (RFS), and log-rank test was used to evaluate statistically significant differences. Causal mediation analysis was performed to investigate the mediation effect of PTBD on OS and RFS.

All statistical analyses were performed in the STATA (version 14), and p < 0.05 was considered statistically significant. The figure was re-arranged in the Adobe Illustrator (version 2019).

## Results

### Basic clinical characteristics of patients accepting PTBD or not

In this study, a total of 341 HCCA patients who accepted radical resection were included. As shown in **Table 1**, the differences of basic epidemiologic features like age, gender, weight, and chronic diseases between the PTBD group and the non-PTBD group were not statistically significant (p > 0.05). In the PTBD group, the number of white blood cells (7.3 vs. 6.3 × 10^9^/L, p = 0.001) and platelets (261.3 vs. 232.5 × 10^9^/L, p = 0.005) was significantly higher than that in the non-PTBD group. Also, levels of bilirubin, including total bilirubin (317.5 vs. 116.6 µmol/L, p = 0.001) and direct bilirubin (167.8 vs. 60.1 µmol/L, p = 0.001), were significantly elevated in the PTBD group, reflecting the presence of preoperative jaundice as a crucial indication for PTBD acceptance. Furthermore, tumor marker CA242 levels were significantly higher in the PTBD group compared to the non-PTBD group (82.3 vs. 42.9 U/mL, p = 0.002), underscoring its diagnostic relevance in PTBD patients.

**Table 1:** Baseline characteristics of HCCA patients accepting PTBD or not.

Variables	Non-PTBD (N = 178)	PTBD (N = 163)	p-value
Epidemiologic features			
Age (years)	58.3 ± 10.1	58.8 ± 10.0	0.652
Gender/male, n (%)	96 (53.9%)	75 (64.1%)	0.087
Weight (kg)	55.1 ± 16.1	55.7 ± 11.7	0.723
CHD, n (%)	8 (4.5%)	2 (1.2%)	0.074
Diabetes, n (%)	14 (7.9%)	15 (9.2%)	0.670
Hypertension, n (%)	28 (15.8%)	18 (11.0%)	0.198
CCI score	1.9 ± 1.3	2.1 ± 1.7	0.069
Laboratory tests			
WBC (×10^9^/L)	6.3 ± 2.2	7.3 ± 2.5	0.001
Platelet (×10^9^/L)	232.5 ± 78.6	261.3 ± 98.8	0.005
Hemoglobin (g/L)	125.2 ± 25.6	122.0 ± 25.8	0.285
ALT (IU/L)	168.9 ± 166.2	145.1 ± 121.8	0.148
AST (IU/L)	155.0 ± 165.8	127.0 ± 102.0	0.076
Total bilirubin (µmol/L)	116.6 ± 87.4	317.5 ± 136.1	0.001
Direct bilirubin (µmol/L)	60.1 ± 52.8	167.8 ± 153.5	0.001
Albumin (g/L)	39.1 ± 4.5	36.4 ± 5.1	0.001
ALP (U/L)	540.7 ± 409.2	565.2 ± 346.5	0.567
γ-GGT (U/L)	771.1 ± 745.5	742.8 ± 1567.5	0.836
PT (s)	19.1 ± 95.5	13.1 ± 11.6	0.447
CEA (µg/L)	6.0 ± 18.2	4.9 ± 10.0	0.500
CA199 (kU/L)	391.5 ± 867.1	494.6 ± 737.2	0.307
CA242 (U/mL)	42.9 ± 79.3	82.3 ± 126.3	0.002
Clinicopathological features			
Liver cirrhosis, n (%)	16 (9.0%)	10 (6.1%)	0.314
Bismuth–Corlette classification, n (%)			
Types III and IV	132 (76.3%)	108 (66.3%)	0.042
Hepatic artery invasion, n (%)	35 (19.8%)	45 (27.6%)	0.089
Portal vein invasion, n (%)	46 (25.8%)	65 (39.9%)	0.006
Positive lymph nodes, n (%)	60 (33.7%)	74 (45.4%)	0.027
Poor differentiation, n (%)	24 (14.1%)	40 (25.5%)	0.010
Endoscopic vascular invasion, n (%)	18 (10.3%)	20 (12.4%)	0.550
Endoscopic nerve invasion, n (%)	76 (43.7%)	74 (46.0%)	0.674
AJCC TNM stage ≥ III, n (%)	89 (50.0%)	103 (63.2%)	0.014
Tumor diameter (cm)	5.7 ± 18.9	4.7 ± 16.7	0.552

AJCC TNM, American Joint Committee on Cancer Tumor Node Metastasis; ALP, Alkaline phosphatase; ALT, Alanine aminotransferase; AST, Aspartate aminotransferase; CA199, Carbohydrate antigen 199; CA242, Carbohydrate antigen 242; CEA, Carcinoembryonic antigen; CCI, Charlson comorbidity index; CHD, Coronary heart disease; γ-GGT, γ-glutamyltransferase; PT, Prothrombin time; WBC, White blood cell.

Particularly, the proportion of patients with portal vein invasion (39.9% vs. 25.8%, p = 0.006) and lymph node metastasis (45.4% vs. 33.7%, p = 0.027) was significantly higher in the PTBD group compared to that in the non-PTBD group. Additionally, there were more patients with poorly differentiated tumors (25.5% vs. 14.1%, p = 0.010) in the PTBD group than that in the non-PTBD group.

### Patients accepting PTBD experienced higher risk for portal vein invasion

To evaluate potential preoperative risk factors associated with portal vein invasion, univariate analysis was conducted initially (**Table 2**). Significant factors identified included liver cirrhosis (OR = 4.4, P = 0.001), total bilirubin > 42 µmol/L (OR = 2.1, P = 0.036), Alanine aminotransferase (ALT) > 42 IU/L (OR = 2.2, P = 0.032), Alanine aminotransferase (AST) > 42 IU/L (OR = 2.8, P = 0.018), Alanine aminotransferase (ALP) > 188 U/L (OR = 4.9, P = 0.009), PTBD (OR = 1.9, P = 0.006), and Bismuth–Corlette type III–IV classification (OR = 2.4, P = 0.002). These factors were found to be statistically significant in association with portal vein invasion. Afterward, these factors with p-values less than 0.1 in univariate analysis and those considered clinically important were included in the multivariable logistic regression. As shown in **Table 2**, previous PTBD (OR = 2.3, P = 0.002) was an independent risk factor for portal vein invasion.

**Table 2:** Univariable and multivariable analysis of risk factors associated with portal vein invasion.

Factors	Univariable analysis		Multivariable analysis	
	OR (95% CI)	p-value	OR (95% CI)	p-value
Age > 60	0.765 (0.485–1.205)	0.247	0.714 (0.420–1.214)	0.213
Male	1.551 (0.971–2.477)	0.066	1.412 (0.824–2.418)	0.209
Liver cirrhosis	4.421 (1.902–10.274)	0.001	5.351 (2.021–14.170)	0.001
WBC > 9.5 (10**^9^**/L)	0.873 (0.482–1.580)	0.654		
Platelet > 300 (10**^9^**/L)	0.950 (0.582–1.550)	0.836		
Total bilirubin > 42 (µmol/L)	2.086 (1.050–4.143)	0.036		
Albumin < 30 (g/L)	0.505 (0.139–1.826)	0.297		
ALT > 42 (IU/L)	2.169 (1.067–4.409)	0.032	1.319 (0.494–3.523)	0.581
AST > 42 (IU/L)	2.781 (1.191–6.493)	0.018	1.623 (0.518–5.084)	0.406
ALP > 188 (U/L)	4.990 (1.483–16.790)	0.009		
γ-GGT > 150 (U/L)	0.997 (0.514–1.934)	0.993		
CA199 > 35 (U/mL)	2.144 (0.908–5.059)	0.082	1.850 (0.724–4.727)	0.199
CEA > 5 (µg/L)	1.143 (0.704–1.855)	0.589		
CA242 > 20 (IU/mL)	1.108 (0.700–1.756)	0.661		
PTBD	1.903 (1.202–3.013)	0.006	2.337 (1.361–4.013)	0.002
Bismuth–Corlette classification (≥ III)	2.389 (1.359–4.197)	0.002	3.925 (1.991–7.736)	0.001
Tumor diameter > 2.5 (cm)	1.404 (0.882–2.233)	0.153		

ALP, Alkaline phosphatase; ALT, Alanine aminotransferase; AST, Aspartate aminotransferase; CA199, Carbohydrate antigen 199; CA242, Carbohydrate antigen 242; CEA, Carcinoembryonic antigen; γ-GGT, γ-glutamyl transpeptidase.

In the univariate analysis, both total bilirubin and PTBD showed statistically significant differences. However, the level of total bilirubin was excluded from the multivariate analysis because it was a key determinant for deciding PTBD, which was central and critical to this study. Including both variables could introduce confounding effects (14). This approach helped avoid multicollinearity and ensured the clarity of PTBD’s impact on outcomes (15).

### Patients accepting PTBD experienced higher risk for lymph node metastasis

Similarly, univariate and multivariate logistic regression analyses were performed to explore the potential relationships between these peri-operative factors and lymph node metastasis, with the results presented in **Table 3**. In the univariate analysis, PTBD (OR = 1.6, P = 0.028) and tumor diameter > 2.5 cm (OR = 1.7, P = 0.016) were the only two factors significantly associated with lymph node metastasis. Afterward, these factors with p-values less than 0.1 in univariate analysis and those considered clinically important were included in the multivariable logistic regression. Following multivariate analysis, CA242 > 20 IU/mL (OR = 1.9, P = 0.016), PTBD (OR = 1.9, P = 0.008), and tumor diameter > 2.5 cm (OR = 1.8, P = 0.022) emerged as three independent risk factors associated with lymph node metastasis.

**Table 3:** Univariable and multivariable analysis of risk factors associated with lymph nodes metastasis.

Factors	Univariable analysis		Multivariable analysis	
	OR (95% CI)	p-value	OR (95% CI)	p-value
Age > 60	0.805 (0.521–1.244)	0.328	0.775 (0.482–1.247)	0.293
Male	1.083 (0.697–1.683)	0.722		
Liver cirrhosis	1.886 (0.844–4.214)	0.122	1.988 (0.824–4.791)	0.126
WBC > 9.5 (10**^9^**/L)	0.795 (0.449–1.407)	0.432		
Platelet > 300 (10**^9^**/L)	0.889 (0.555–1.424)	0.625		
Total bilirubin > 42 (µmol/L)	0.975 (0.542–1.753)	0.932		
Albumin < 30 (g/L)	1.031 (0.358–2.967)	0.954		
ALT > 42 (IU/L)	1.270 (0.688–2.343)	0.444		
AST > 42 (IU/L)	0.911 (0.474–1.753)	0.781		
ALP > 188 (U/L)	1.652 (0.736–3.706)	0.224		
γ-GGT > 150 (U/L)	1.250 (0.653–2.394)	0.501		
CA199 > 35 (U/mL)	0.896 (0.444–1.806)	0.758	0.567 (0.245–1.311)	0.185
CEA > 5 (µg/L)	0.889 (0.555–1.424)	0.625		
CA242 > 20 (IU/mL)	1.550 (0.992–2.422)	0.055	1.934 (1.130–3.309)	0.016
PTBD	1.635 (1.055–2.533)	0.028	1.936 (1.189–3.153)	0.008
Bismuth–Corlette classification (≥ III)	1.550 (0.941–2.551)	0.085	1.463 (0.842–2.543)	0.177
Tumor diameter > 2.5 (cm)	1.732 (1.106–2.713)	0.016	1.793 (1.087–2.960)	0.022

ALP, Alkaline phosphatase; ALT, Alanine aminotransferase; AST, Aspartate aminotransferase; CA199, Carbohydrate antigen 199; CA242, Carbohydrate antigen 242; CEA, Carcinoembryonic antigen; γ-GGT, γ-glutamyl transpeptidase.

### Survival analysis

**Figure 1:**
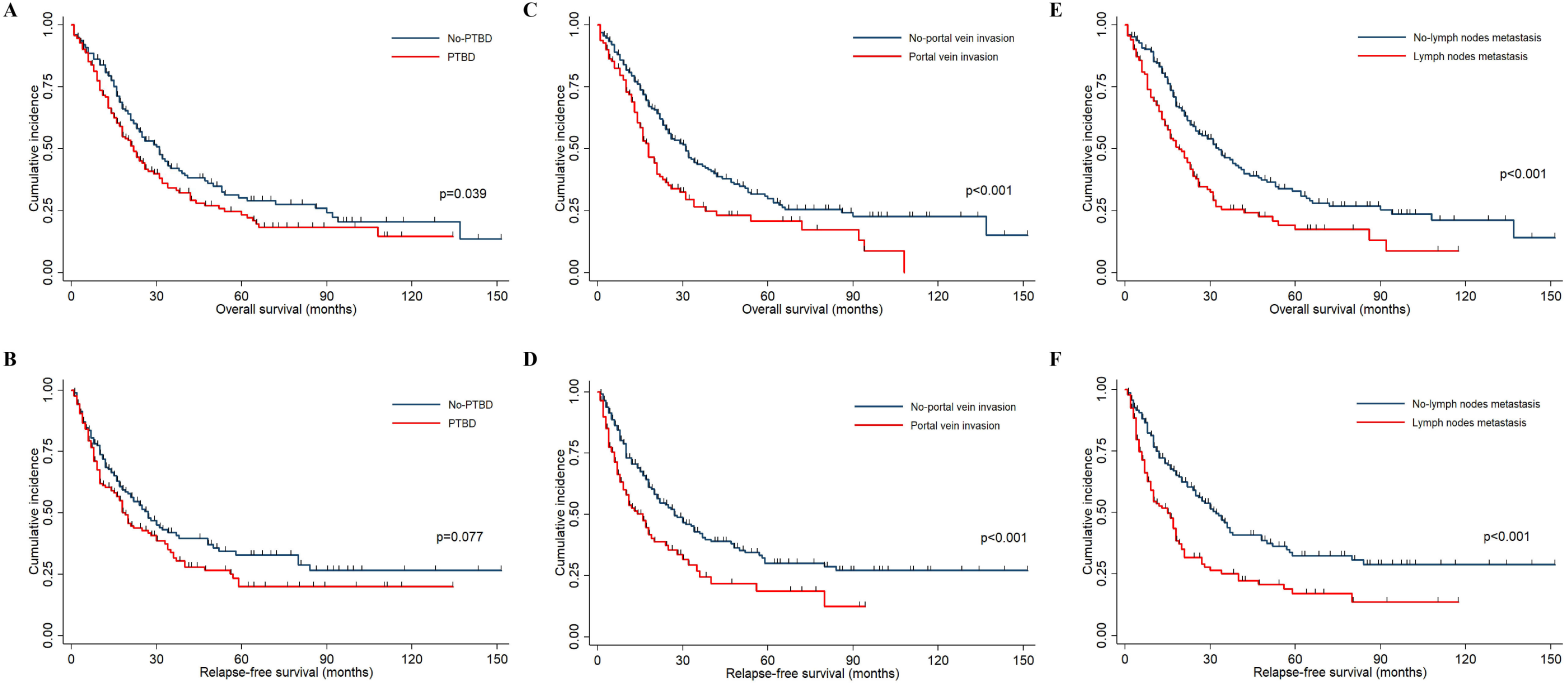
The Kaplan–Meier curves indicated the OS and RFS in HCCA patients. **(A, B)** The OS and RFS survival curves between patients accepting PTBD or not. **(C, D)** The OS and RFS survival curves between patients with and without portal vein invasion. **(E, F)** The OS and RFS survival curves between patients with and without lymph node metastasis.

This study aimed to investigate the relationship between preoperative percutaneous transhepatic biliary drainage (PTBD), portal vein invasion, lymph node metastasis, and clinical survival in patients with hilar cholangiocarcinoma. The Kaplan–Meier curves were drawn, and the log-rank test was used to evaluate differences between two groups. As presented in **Figures 1A, B**, these patients who did not accept preoperative PTBD showed a significantly longer OS (p = 0.039) than the PTBD group, whereas the RFS did not show a statistical difference between two groups (p = 0.082). Consistent with other studies, those patients without portal vein invasion and lymph node metastasis had a significantly better prognosis both in OS and RFS (all p < 0.001), which was shown in **Figures 1C–F**.

Furthermore, univariate and multivariate Cox regression models were employed to investigate potential risk factors associated with OS and RFS. Although preoperative PTBD emerged as a significant risk factor for OS (HR = 1.3, P = 0.039) in the univariate analysis, this association did not retain statistical significance (HR = 0.99, P = 0.919) in the multivariate analysis (**Table 4**). Moreover, the preoperative PTBD was not directly related to RFS in the univariate and multivariate analysis (**Table 5**). However, the portal vein invasion and lymph node metastasis were two significant risk factors associated with OS and RFS (all p < 0.01). Subsequently, causal mediation analysis was performed to investigate the mediation effect of PTBD on OS and RFS. The analysis revealed that portal vein invasion accounted for 30.1% (p = 0.030) of PTBD’s effect on OS and 36.4% (p = 0.022) on RFS. Similarly, lymph node metastasis mediated 35.9% (p = 0.038) of PTBD’s effect on OS and 18.1% (p = 0.034) on RFS, respectively. These findings indicated that the effect of PTBD on survival was partly mediated by portal vein invasion and lymph node metastasis.

**Table 4:** Univariable and multivariable analysis of risk factors associated with overall survival.

Factors	Univariable analysis		Multivariable analysis	
	HR (95% CI)	p-value	HR (95% CI)	p-value
Age > 60	1.181 (0.904–1.543)	0.223		
Male	1.174 (0.892–1.544)	0.253		
BMI ≥ 18.5	0.941 (0.553–1.601)	0.823		
HBV	1.172 (0.721–1.905)	0.522		
CA199 > 35 (U/mL)	1.485 (0.951–2.318)	0.082	1.035 (0.635–1.688)	0.889
CA242 > 20 (IU/mL)	1.603 (1.214–2.117)	0.001	1.394 (1.021–1.902)	0.036
CEA > 5 (µg/L)	1.125 (0.843–1.501)	0.425		
PTBD	1.325 (1.014–1.733)	0.039	0.985 (0.742–1.308)	0.919
Bismuth–Corlette classification (≥ III)	0.872 (0.644–1.180)	0.374		
Tumor diameter > 2.5 (cm)	1.186 (0.899–1.563)	0.227		
Poor tumor differentiation	1.699 (1.210–2.386)	0.002	1.643 (1.167–2.314)	0.004
Portal vein invasion	1.610 (1.216–2.131)	0.001	1.473 (1.099–1.975)	0.010
Hepatic artery invasion	1.556 (1.138–2.128)	0.006	1.211 (0.873–1.681)	0.252
Lymph nodes metastasis	1.688 (1.286–2.215)	0.001	1.617 (1.217–2.148)	0.001
Microscopic nerve invasion	0.867 (0.657–1.145)	0.315		

BMI, Body Mass Index; CA199, Carbohydrate antigen 199; CA242, Carbohydrate antigen 242; CEA, Carcinoembryonic antigen.

**Table 5:** Univariable and multivariable analysis of risk factors associated with relapse-free survival.

Factors	Univariable analysis		Multivariable analysis	
	HR (95% CI)	p-value	HR (95% CI)	p-value
Age > 60	0.884 (0.666–1.174)	0.395		
Male	1.105 (0.829–1.474)	0.495		
BMI ≥ 18.5	1.281 (0.693–2.369)	0.429		
HBV	1.359 (0.834–2.215)	0.218		
CA199 > 35 (U/mL)	1.455 (0.903–2.347)	0.124		
CA242 > 20 (IU/mL)	1.499 (1.120–2.007)	0.007	1.328 (0.978–1.803)	0.070
CEA > 5 (µg/L)	0.936 (0.684–1.279)	0.677		
PTBD	1.285 (0.969–1.705)	0.082	0.945 (0.702–1.274)	0.712
Bismuth–Corlette classification (≥ III)	1.011 (0.731–1.400)	0.945		
Tumor diameter > 2.5 (cm)	0.990 (0.753–1.334)	0.949		
Poor tumor differentiation	1.265 (0.865–1.847)	0.224		
Portal vein invasion	1.687 (1.252–2.273)	0.001	1.546 (1.128–2.118)	0.007
Hepatic artery invasion	1.541 (1.104–2.150)	0.011	1.276 (0.898–1.813)	0.174
Lymph nodes metastasis	1.879 (1.413–2.499)	0.001	1.855 (1.380–2.492)	0.001
Microscopic nerve invasion	0.760 (0.566–1.020)	0.068	0.734 (0.545–0.990)	0.043

BMI, Body Mass Index; CA199, Carbohydrate antigen 199; CA242, Carbohydrate antigen 242; CEA, Carcinoembryonic antigen; HBV, Hepatitis B virus.

### Postoperative complications in HCCA patients accepting PTBD or not

In HCCA patients, the presence of postoperative complications was an important factor affecting hospital mortality and long-term survival (16, 17). In this study, the comparison of postoperative complications is between the PTBD group and the non-PTBD group, and the results were presented in **Table 6**. The proportion of pleural effusion (28.0% vs. 14.6%, p = 0.004) in the PTBD group was significantly higher than that in the non-PTBD group. Additionally, the mean length of hospitalization was 26.7 days, which was obviously longer than that in the non-PTBD group (21.8 days, p = 0.002). The presence of other complications including fever, infection, bile leakage, cholangitis, liver failure, and bleeding was similar in the PTBD group and the non-PTBD group.

**Table 6:** Comparison of post-operative complications between HCCA patients accepting PTBD or not.

Variables	Non-PTBD (N = 178)	PTBD (N = 163)	p-value
Fever, n (%)	59 (36.9%)	66 (41.5%)	0.397
Infection, n (%)	86 (53.8%)	95 (59.8%)	0.280
Bile leakage, n (%)	23 (14.5%)	31 (19.5%)	0.232
Ascites, n (%)	19 (12.1%)	28 (18.1%)	0.141
Pleural effusion, n (%)	22 (14.6%)	42 (28.0%)	0.004
Cholangitis, n (%)	5 (3.14%)	12 (7.59%)	0.079
Liver failure, n (%)	6 (3.77%)	5 (3.14%)	0.759
Bleeding, n (%)	21 (13.2%)	23 (14.6%)	0.728
Blood transfusion, n (%)	58 (38.9%)	57 (37.5%)	0.799
Additional operation, n (%)	10 (6.33%)	8 (5.03%)	0.618
Length of hospitalization (days)	21.8 ± 12.1	26.7 ± 14.5	0.002
Severe complication, n (%) (Clavien–Dindo ≥ III)	42 (23.6%)	27 (16.6%)	0.106

## Discussion

In this study, we demonstrated that patients with HCCA who underwent preoperative PTBD exhibited a significantly higher incidence of portal vein invasion and regional lymph node metastasis compared to those who did not undergo PTBD. These differences were statistically significant, highlighting a potential adverse effect of PTBD on tumor local spread.

According to previous studies, several mechanisms might explain why PTBD could lead to increased local tumor metastasis. Firstly, the PTBD procedure itself might cause mechanical injury to the tumor or surrounding tissues like branches of vessels, potentially dislodging tumor cells (18). These dislodged cells could then enter the bloodstream or lymphatic system, facilitating metastasis (19, 20). Moreover, the PTBD might induce a local inflammatory response, which could create a microenvironment that supports tumor growth and spread. Inflammation might suppress local immune responses and promote angiogenesis, providing tumor cells with nutrients and pathways for dissemination (21, 22). Furthermore, prolonged intervals between PTBD and definitive surgery might also increase risks of local tumor progression, including vascular invasion and lymph node metastasis.

Although PTBD in HCCA patients increased the risk of portal vein invasion and lymph node metastasis, the Cox regression model indicated that PTBD was not an independent risk factor for OS and RFS, and the survival curves showed no statistically significant difference in RFS between the PTBD and non-PTBD groups. Notably, patients with portal vein invasion and lymph node metastasis had significantly lower survival rates compared to those without these invasions. This paradoxical finding might be explained through the concept of causal mediation effects. PTBD may indirectly affect survival by increasing the likelihood of portal vein invasion and lymph node metastasis, which were strong prognostic factors for reduced survival. However, other variables like serum elevated markers influencing portal vein invasion and lymph node metastasis may still exist, potentially diluting the direct impact of PTBD on overall survival (23).

The findings of this study might have significant clinical implications for clinicians, as they may need to re-assess the use of PTBD in the preoperative management of HCCA patients. Although PTBD was effective in reducing jaundice and improving liver function, its potential to facilitate local spread of tumor cells must be carefully weighed. Secondly, identifying which patients would most benefit from PTBD and considering the risks of enhanced metastasis were crucial. High-risk patients might need alternative approaches [including endoscopic nasobiliary drainage (ENBD) or endoscopic biliary stenting (EBS)] or closer monitoring for signs of metastasis. Additionally, the safety of the PTBD procedure must be mentioned, as portal vein injury during this intervention was already reported (24). The potential spread of tumor cells from the bile duct into the portal vein system might cause portal vein invasion and micro-metastasis throughout the liver.

However, this study also had several limitations that should be acknowledged. Firstly, as a retrospective clinical study, we could not provide exact mechanisms how the PTBD increased in the incidence of portal vein invasion and lymph node metastasis. The subsequent basic biological studies needed to be conducted. Secondly, the extremely limited number of patients undergoing preoperative ENBD or EBS for biliary drainage in our cohort precluded meaningful comparisons of their effects on lymph node metastasis or portal vein invasion. This limitation narrowed the generalizability of our findings, as the relative safety profiles and tumor progression risks associated with different biliary drainage strategies remained incompletely addressed. Future multicenter studies with larger patient cohorts accepting diverse drainage approaches were needed to verify and extend these findings.

## Conclusion

In conclusion, our study indicated that PTBD might be associated with an increased risk of local tumor metastasis including portal vein invasion and lymph node metastasis in HCCA patients. Except for needle tract tumor seeding during the PTBD, its potential to facilitate regional tumor spread warranted careful consideration in clinical decision-making. Further research is necessary to fully understand the implications of PTBD and to develop strategies that optimize patient outcomes.

## Data Availability

The raw data supporting the conclusions of this article will be made available by the authors, without undue reservation.
